# Supplementary material for: An alveolus lung-on-a-chip model of Mycobacterium fortuitum lung infection
Source: Dis Model Mech. 2025 Aug 1;18(9):dmm052085. doi: 10.1242/dmm.052085 (PMC12590473; doi:10.1242/dmm.052085)
Supplement: Supplementary information [file dmm-18-052085-s1.pdf]

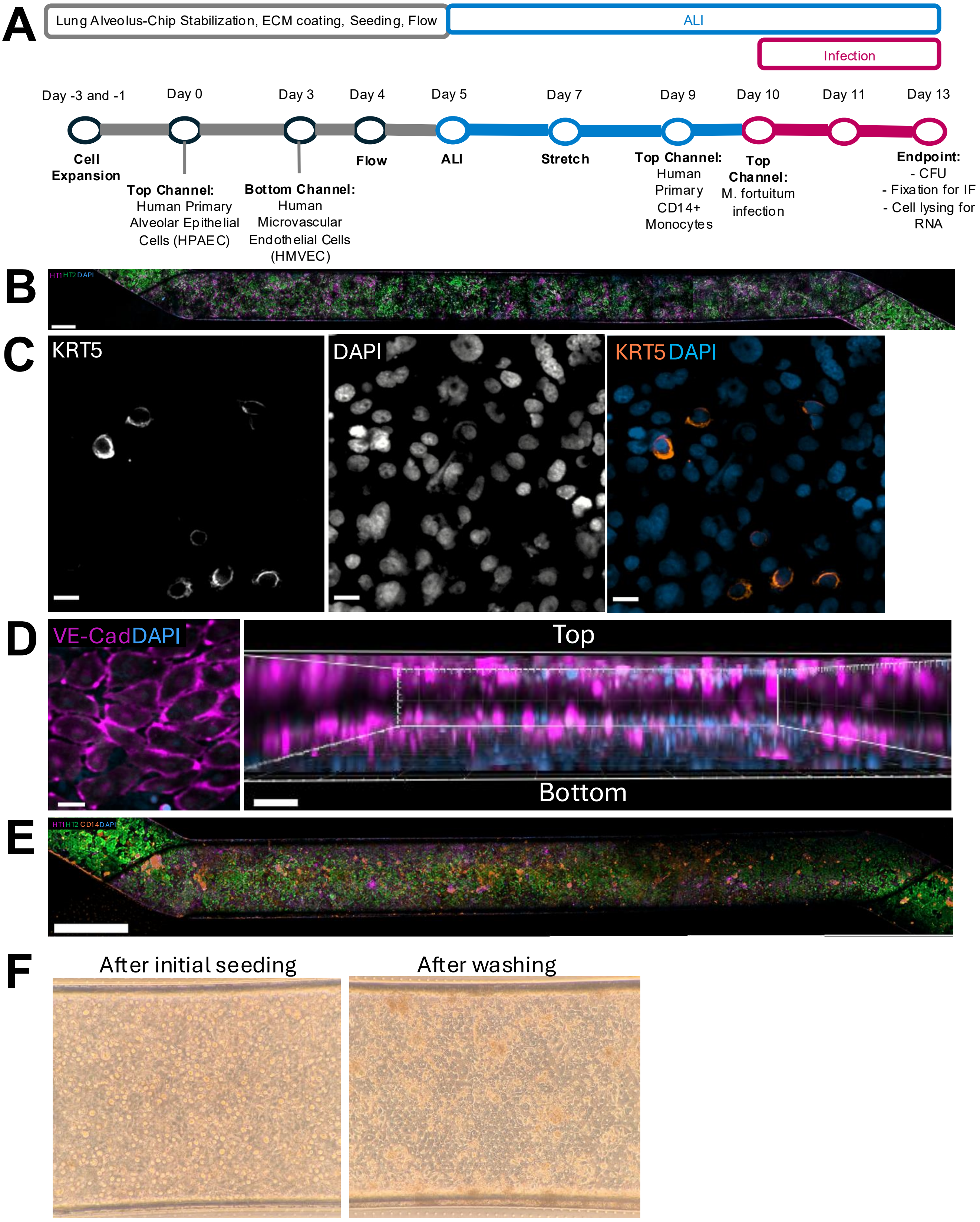

A
Lung Alveolus-Chip Stabilization, ECM coating, Seeding, Flow
ALI
Infection
Day -3 and -1
Day 0
Day 3
Day 4
Day 5
Day 7
Day 9
Day 10
Day 11
Day 13
Cell Expansion
Top Channel: Human Primary Alveolar Epithelial Cells (HPAEC)
Bottom Channel: Human Microvascular Endothelial Cells (HMVEC)
Flow
ALI
Stretch
Top Channel: Human Primary CD14+ Monocytes
Top Channel: M. fortuitum infection
Endpoint: - CFU - Fixation for IF - Cell lysing for RNA
B
C
KRT5
DAPI
KRT5 DAPI
D
VE-Cad DAPI
Top
Bottom
E
F
After initial seeding
After washing

Fig. S1. Establishment of the alveolus lung-on-a-chip model. A. Typical timeline of a single alveolus lung-on-a-chip (ALoC) experiment. **B.** Stitched image of the entire apical channel containing AT1 (HT1+, magenta) and AT2 cells (HT2+, green). Nuclei are labelled with DAPI (blue). Scale bar = 500 µm. A magnified region of this image is presented in Fig. 1C. **C.** A single z-slice image highlighting KRT5+ cells (orange) on an uninfected ALoC. Nuclei are labelled with DAPI (blue). Scale bar = 50 µm. **D.** A single z-slice image of the bottom of the basal compartment of an uninfected ALoC containing macrophages. Human lung microvascular endothelial cells (HMVEC) are labelled with VE-cadherin (magenta). Nuclei are labelled with DAPI. Scale bar = 50 µm. **E.** Stitched image of the entire apical channel of an ALoC containing macrophages (CD14+, orange), AT1 cells (HT1+, magenta), and AT2 cells (HT2+, green). Nuclei are labelled DAPI (blue). Scale bar = 500 µm. **F.** Brightfield image of an uninfected ALoC immediately after the addition human CD14+ peripheral blood-derived macrophages (macrophages) on top of the alveolar epithelial cells **(left)**. Brightfield image of an uninfected ALoC 24 hours after the addition of macrophages **(right)**.

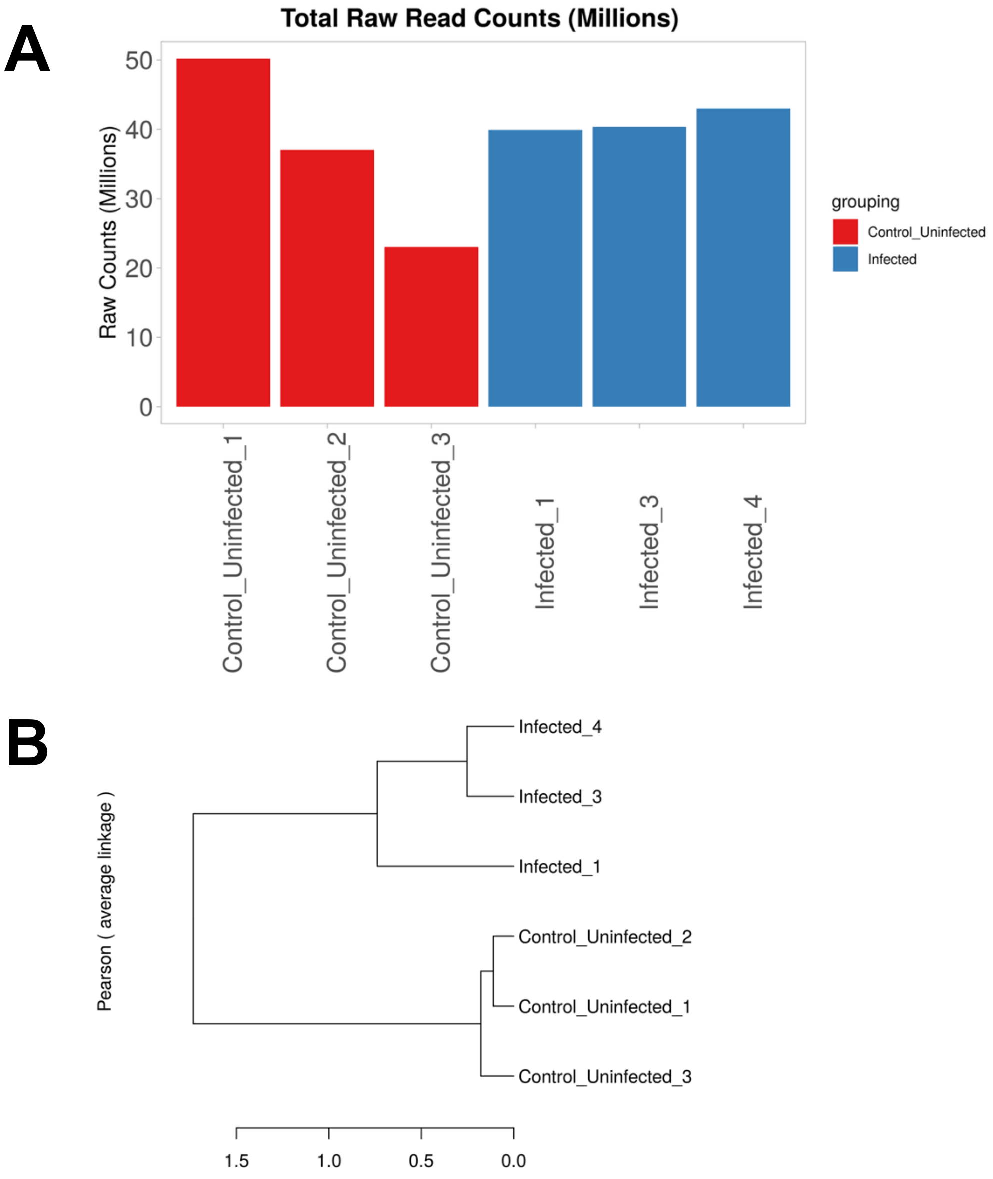

Fig. S2. Bulk RNA-sequencing analysis of *M. fortuitum*-infected ALoCs A. Total raw read counts of uninfected (n=3) and *M. fortuitum*-infected (n=3) ALoCs. **B.** Pearson (average linkage) clustering of *M. fortuitum*-infected ALoCs and uninfected ALoCs.

Disease Models & Mechanisms • Supplementary information

Table S1. Antibodies used on ALoC for immunofluorescence

Cell Type	Target	Primary Antibody	Secondary Antibody
**AT1**	HT1	Anti HT1-56 (Terrace Biotech, TB-29AHT1-56)	Alpaca anti-Mouse IgG1 Nano (VHH) Recombinant Secondary Antibody conjugated with Alexa Fluor 647 (Thermo Fisher, SA5-10333)
**AT2**	HT2	Anti HT2-280 (Terrace Biotech, TB-27AHT2-280)	Goat anti-Mouse IgG2a Cross-Adsorbed Secondary Antibody, Alexa Fluor 488 (Thermo Fisher, A21131)
**Macrophage**	CD14	CD14 Recombinant Rabbit Monoclonal Antibody (SC69-02) (Thermo Fisher, MA5-32248)	Donkey anti-Rabbit IgG (H+L), highly cross-adsorbed Secondary Antibody with Alexa Fluor 555 Plus
**Epithelial cell**	E-cadherin	E-cadherin recombinant rabbit monoclonal antibody (5H6L18)	Donkey anti-Rabbit IgG (H+L), highly cross-adsorbed Secondary Antibody with Alexa Fluor 555 Plus
**Endothelial cell**	VE-cadherin	CD144 (VE-cadherin) Monoclonal Antibody (16B1), eBioscience (Invitrogen 14-1449-82)	Alpaca anti-Mouse IgG1 Nano (VHH) Recombinant Secondary Antibody conjugated with Alexa Fluor 647 (Thermo Fisher, SA5-10333)
**n/a**	Nuclei	NucBlue™ Fixed Cell ReadyProbes™ Reagent (DAPI) (Thermo Fisher, R37606)	n/a

Disease Models & Mechanisms • Supplementary information
